# Hydrogels and Cell Based Therapies in Spinal Cord Injury Regeneration

**DOI:** 10.1155/2015/948040

**Published:** 2015-06-01

**Authors:** Rita C. Assunção-Silva, Eduardo D. Gomes, Nuno Sousa, Nuno A. Silva, António J. Salgado

**Affiliations:** ^1^Life and Health Sciences Research Institute (ICVS), School of Health Sciences, University of Minho, Campus de Gualtar, 4710-057 Braga, Portugal; ^2^ICVS/3B's, PT Government Associate Laboratory, Braga/Guimarães, Portugal

## Abstract

Spinal cord injury (SCI) is a central nervous system- (CNS-) related disorder for which there is yet no successful treatment. Within the past several years, cell-based therapies have been explored for SCI repair, including the use of pluripotent human stem cells, and a number of adult-derived stem and mature cells such as mesenchymal stem cells, olfactory ensheathing cells, and Schwann cells. Although promising, cell transplantation is often overturned by the poor cell survival in the treatment of spinal cord injuries. Alternatively, the therapeutic role of different cells has been used in tissue engineering approaches by engrafting cells with biomaterials. The latter have the advantages of physically mimicking the CNS tissue, while promoting a more permissive environment for cell survival, growth, and differentiation. The roles of both cell- and biomaterial-based therapies as single therapeutic approaches for SCI repair will be discussed in this review. Moreover, as the multifactorial inhibitory environment of a SCI suggests that combinatorial approaches would be more effective, the importance of using biomaterials as cell carriers will be herein highlighted, as well as the recent advances and achievements of these promising tools for neural tissue regeneration.

## 1. Introduction

SCI is a devastating condition that often leads to permanent functional and neurological deficits in injured individuals. The limited ability of the CNS to spontaneously regenerate, mainly due to the establishment of an inhibitory environment around the lesion site and to the formation of a dense scar tissue, impairs axonal regeneration and functional recovery of the spinal cord [1–3].

The annual incidence of SCI has been reported to be 25.5 cases per million [4], at an average age of 31.7 years [5]. Moreover, its prevalence ranges from 236 per million in India to 1800 per million in USA [6]. The leading causes of SCI are motor-vehicle crashes, sports-associated accidents, falls, and violence-related injuries [7].

The severity of an injury is accurately conveyed by the five-level (A–E) American Spinal Injury Association (ASIA) Impairment Scale (AIS). Upon evaluation of the severity of the damage, the lesion is broadly characterized as complete or incomplete [8, 9], with distinct clinical implications to the patients (e.g., paralysis, sensory loss, intractable pain, pressure sores, and urinary/other infections) [5, 8]. This generates tremendous emotional, economic, and social repercussions for the patients and their families.

The aggressive pathophysiology of SCI contributes to the extension of this debilitating condition. A mechanical trauma to the spinal cord triggers an immediate cascade of cellular and biochemical events that contribute to the progression of the lesion. Blood vessels disruption and extensive cell death are some posttraumatic changes that result from the primary injury [1, 10]. In response to this, a set of secondary events occur. An inflammatory environment is established by macrophages, neutrophils, and leukocytes, which are recruited in order to phagocyte cell debris and prevent further uncontrolled tissue damage [3, 11, 12]. From days to weeks, a fluid-filled cyst is formed at the injury site, surrounded by a glial scar mainly constituted by reactive astrocytes. These cells secrete several inhibitory proteins such as chondroitin sulfate proteoglycans (GSPGs) and axonal growth inhibitors [12, 13], thus preventing axonal regeneration and remyelination along the spinal cord. Even though the role of the glial scar is to stabilize and ultimately protect the damaged spinal cord, it largely incapacitates spinal cord long-distance functional regeneration [14], leading to the establishment of a chronic injury.

Unfortunately, there is still no effective clinical treatment for SCI, besides some clinical attempts to provide recovery to patients. As recently reviewed by Silva et al. [14], the most usual procedures rely on surgical techniques, including surgical decompression and further stabilization of the spine, as well as on pharmacological interventions. Several pharmacological agents have been studied in this context [15], high dose methylprednisolone (MP) administration being an option for the treatment of acute SCI. However, its efficacy is quite limited due to severe side effects [14, 16]. Therefore it is recommended to be given to patients only with the knowledge that evidence suggesting harmful side effects is more consistent than any possible clinical benefits [17].

In recent years, tissue engineering and regenerative medicine based approaches have been proposed as alternatives for SCI repair/regeneration. For the past decades, cell-based therapies have been highlighted for SCI regeneration [18], as well as engineering approaches using biomaterials. Nowadays, the combination of biomaterials with cell transplantation is also being widely explored in the scope of SCI. In this context, biomaterials are expected to stabilize the lesion site, while directly delivering the cells into it, and provide an adequate environment for the regeneration of the injured tissues. Several cell types and biomaterials have been suggested for the development of promising regenerative strategies for SCI. Therefore, the aim of this review is to address the recent progress that has been made in both approaches. A discussion on the potential of these therapies for SCI regeneration will be the starting point, after which the contributions of biomaterials for the development of more efficient cell-based therapies will be also discussed.

## 2. Cell-Based Therapies for SCI Repair 

Aiming at developing successful therapies for SCI treatment, the transplantation of certain cell populations into damaged areas has been one of the most used regenerative approaches over the years. Among the alternatives, stem-cell based transplantation has been gathering attention for the past 15 years [19–23]. Most of the times stem cells are used because of their differentiation potential [24–26]; however they have been also shown to be able to provide a large repertoire of signaling molecules, including anti-inflammatory cytokines and growth factors. These may modulate the inhibitory environment of SCI while increasing the trophic support to resident cells [27–32]. So far, stem cells from different origins have been tested for their ability to stimulate nerve regeneration and restore the neuronal circuitry when integrated in the injured site [14, 33].

### 2.1. Embryonic Stem Cells

One of the cell populations proposed for SCI regeneration is embryonic stem cells (ESCs) [34], which are known to differentiate into all fetal cell lineages [24], thus being considered as pluripotent.

The ability of ESCs to differentiate into neural and glial cells in* in vitro* culture systems has been extensively explored using different strategies. Retinoic acid (RA) and embryoid body- (EB-) based protocols have been used to induce neural differentiation of ESCs in culture, resulting in the activation of a complex system of neuronal gene expression provided by neuronal like cells [35] and in the production of oligodendrocytes, capable of producing myelin for the myelination of neurons in culture [19]. Another approach, consisting in the use of specific factors in mouse ESCs culture, was found to efficiently direct cell differentiation into dopaminergic and serotonergic neurons [36, 37]. The use of cell culture media specifically defined for ESCs commitment to the neural fate is also an alternative method [38]. Of particular interest is the possibility to genetically modify the ESCs, in order to obtain neuronal precursors-enriched cultures [38, 39].

The suitability of ESCs-based approaches for SCI treatment has also been investigated in a number of spinal cord injury models. Keirstead et al. [34] transplanted neural stem cells (NSCs) obtained from mouse ESCs into a rat spinal cord, after an induced thoracic SCI. Most transplanted cells survived, migrated away from the injury site, and were shown to preferentially differentiate into oligodendrocytes and astrocytes [34]. Still, induced ESC-derived oligodendrocyte progenitor cells transplanted into demyelinated spinal cords were found to contribute to the remyelination of host axons. In the same report, the improvement of animals motor performance upon transplantation was also described [19]. Finally, ESC clinical applications in SCI patients started through a Phase I clinical trial provided by Geron's company in 2011. A cohort of patients with complete subacute thoracic SCI was transplanted with predifferentiated oligodendrocyte precursor cells derived from human ESCs for safety studies. Unfortunately, Geron's program was aborted later in that year [40]. Nevertheless, to date no safety issues were reported in five patients submitted to ESCs transplants.

### 2.2. Induced Pluripotent Stem Cells

Recently, another type of pluripotent stem cells, known as induced pluripotent stem cells (iPS cells or iPSCs), emerged as a possible alternative to obtain stem cells directly from adult tissues for autologous transplantation. The iPSCs technology resulted from a pioneer work developed by Yamanaka's lab in Japan in 2006, which showed that the introduction of four transcription factors reverted the phenotype of differentiated adult cells into pluripotent stem cells [41]. iPSCs are often compared to ESCs, as they share similar characteristics, such as pluripotency, self-renewal capacity, and gene expression [42, 43]. Moreover, the potential to acquire abnormal karyotypes and genetic amplification associated with teratoma formation is also a common feature between the two cell types [42, 43]. However, iPSCs differentiation into neural lineages occurs at a lower frequency than for ESCs [44].

The fact that iPSCs can be derived directly from adult tissues offers an unlimited supply of autologous cells, which could be used to generate transplants without the risk of immune rejection. However, safety issues such as those related to tumor formation should be determined prior to their clinical application. Therefore, it is crucial to carefully test iPSCs for tumorigenicity [42, 45]. In line with this, Zhao et al. [21] presented a study concerning the immunogenicity of iPS cells* in vivo*. A teratoma formation assay was used to show that iPSCs efficiently formed teratomas in mice, with a strong immune-rejection of the cells [21]. Later in 2013, Araki et al. [46] attempted to reproduce the conclusions obtained by Zhao and colleagues using a different procedure. By transplanting cells from a chimera obtained from iPSCs clones and a mouse embryo into mice, little or no immunogenic response was observed [46].

Although these recent reports have emphasized the pitfalls of iPSCs technology, others supporting the efficacy of iPSCs as cellular systems for SCI treatment are also accumulating. For example, human iPSC-derived neurospheres (hiPSC-NSs) survived, migrated, and differentiated into the three major neural lineages after transplantation into a nonobese diabetic-severe combined immunodeficient (NOD-SCID) SCI model mice. The formation of synapses between grafted cells and host mouse neurons was promoted, as well as the expression of neurotrophic factors, angiogenesis, axonal regrowth, and myelination in the injured area. As a result, there was an improvement of the functional activity of the hiPSC-NSs-grafted mice, with no tumor formation [47]. More recently, a preclinical study investigated the therapeutic potential of transplanting preevaluated neural stem/progenitor cells (NS/PCs) clones derived from murine and human iPSCs (iPSC-NS/PCs) into a nonhuman primate model of contusive SCI [26]. Similarly to previous studies, the grafted cells were found to survive and differentiate into neurons, astrocytes, and oligodendrocytes, without evidence of tumor formation. In addition, there was an enhancement in axonal sparing/regrowth and angiogenesis at the lesion site and the prevention of the lesion epicenter demyelination. At the end of the treatment, a functional recovery of the animal after SCI was observed [26]. Nevertheless, more preclinical studies have yet to be performed, in order to investigate the true potential and safety of iPSCs, before moving to a clinical setting.

### 2.3. Neural Stem Cells

Another cell population with a possible interest for SCI research is adult multipotent NSCs [27], which are particularly appealing due to their CNS origin. These cells have been shown to generate the three main neural cell lineages of the mammalian CNS in culture [25]. Thus they can hypothetically allow the replacement of spinal neurons lost after injury and differentiate towards astrocytes, to restore the nonneuronal milieu of the preinjured spinal cord, or towards oligodendroglia, to allow remyelination [27]. In fact, previous studies have confirmed this theory. The engraftment of NSCs into a SCI model of contused adult rat spinal cord resulted in the production of neurons that migrated long distances rostrally and caudally, with observed functional improvement [48]. In a cervical contusion-induced SCI in primates,* in vitro*-expanded human neural stem progenitor cells (NSPCs) were grafted nine days after injury and were shown to survive and differentiate into the neural lineages. In addition, there was a decrease in the injury cavities extent, as well as a significant increase of the spontaneous motor activity of the transplanted animals [20]. Furthermore, demyelinated axons in NOD-SCID mice with traumatic SCI were remyelinated after transplantation of human CNS cells grown in aggregates (hCNS-SCns). These cells also differentiated into neurons that exhibited the ability of synapse formation with host neurons [49]. More recently, it has been reported that transplantation of fetal NSCs into complete rat spinal cord transection sites led to the formation of ectopic colonies two months after cell engraftment. These colonies were found to disseminate in widespread areas of the host CNS and continuously proliferate in several neural-cell lineage types [23].

In other studies, the NSCs capacity to promote axonal regeneration was related with the secretion of neurotrophic factors [27]. First* in vitro*, and then* in vivo*, intrinsic growth factor production by NSCs was found to support extensive growth of host axons, which are known to be sensitive to these factors [27]. Furthermore, it was observed that the genetic modification of NSCs alters the overall axonal responses. For instance, the induction of neurotrophin-3 (NT-3) production by NSCs has significantly expanded the growth and penetration of host axons along the injury site [27, 50].

The experimental ground work regarding NSCs as cellular-based therapy has shown promise in repairing damaged cells and tissues after SCI and ultimately led to the attempt of applying this therapy to humans. In line with this, Stem Cells Inc. Company (Switzerland) established the world's first clinical trial in spinal cord injured humans using these cells [51]. In 2011, the company initiated a Phase I/II clinical trial designed to assess both safety and preliminary efficacy of a single transplantation of purified fetal human neural stem cells (HuCNS-SC), as a treatment for chronic thoracic SCI, for both complete and incomplete injuries. The study enrolled seven patients with complete injuries (AIS A) and five patients with incomplete injuries (AIS B). The cells were directly injected into their spinal cords, and they were temporarily immunosuppressed. Clinical updates were reported on a total of eight of the 12 patients enrolled in the clinical trial. With regard to AIS A patients, there was significant posttransplant gain in sensory function in four patients up to date. Concerning AIS B subjects, two of three patients had significant gain in sensory perception, the third remaining unaltered [51].

### 2.4. Mesenchymal Stem Cells

In the last decade, mesenchymal stem cells [52] (MSCs) have also been in the forefront of cell-based strategies for SCI regeneration. These cells were first described to be present in the bone marrow by Friedenstein and colleagues [53]. They were mainly characterized by the ability to adhere to plastic in culture, to develop into fibroblastic colony forming cells (CFU-F), and to differentiate into osteoblasts, adipocytes, and chondroblasts* in vitro *[53–55].

Availability is one of the advantages of MSCs comparing with other cells, as they can be found in several tissues [56–59]. In addition, MSCs isolation can be easily performed [60], without rising any ethical or political issues.

**Figure 1:**
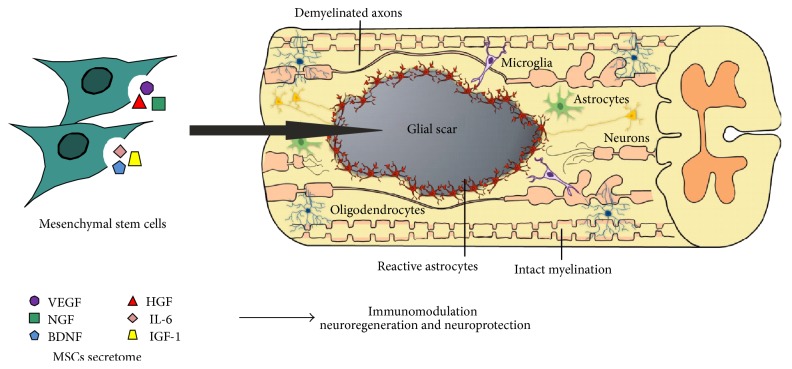
Application of MSCs as a treatment for SCI. The MSCs secretome is believed to be a key player on the promotion of neuroregeneration and neuroprotection, as well as the modulation of the inflammatory response.

The efficacy of MSCs as therapeutic agents for CNS has been related to different theories, starting from their engraftment efficiency when injected into the body [54] to their differentiation into neural phenotypes. The latter was in fact studied both* in vitro*, where bone marrow MSCs (BM-MSCs) were found to putatively differentiate into neuron-like cells and glial cells [52], and* in vivo*, where the authors found BM-MSCs were able to migrate across the blood-brain barrier (BBB^1^), repopulate the CNS, and differentiate into microglia-like cells [61]. Despite these findings, this is still a controversial topic. Indeed, it is more likely that the MSCs potential is associated with their trophic activity [28, 29, 55, 62, 63]. MSCs secrete a set of bioactive molecules and/or microvesicles—their secretome—which is believed to mediate both paracrine and autocrine MSCs activities [29, 62]. In response to injury, the secretome may support the repair and regeneration of damaged tissues by suppressing local immune response [64], enhancing angiogenesis and inhibiting scarring and cell apoptosis [65] (Figure 1).

These outcomes support the multifactorial roles of MSCs transplantation on CNS tissues and cells. Further details on this topic can be found elsewhere [28, 30].

In the context of SCI treatment, different strategies have been considered. While some are solely focused on the transplantation of MSCs in the injury site, others are more interested in the administration of their secretome in the same area in order to support the survival and proliferation of the remaining cells. Regarding the transplantation of MSCs, both intravenous [66–68] and subcutaneous [68] injections have been proposed, as well as a direct injection in the injury site [69]. MSCs transplantation by cell mobilization with granulocyte colony-stimulating factor (G-CSF) [68, 70] or intrathecal catheter delivery [67, 71] was also explored. In all of these studies the authors reported functional recovery after SCI. On the other hand, studies regarding the use of MSCs secretome have also shown promising results. For instance, the conditioned medium (CM) of BM-MSCs promoted the survival and neurite outgrowth of hippocampal neurons* in vitro *[72]. In another study, both adipose stem cells (ASCs) and human umbilical cord perivascular cells (HUCPVCs) CMs were shown to increase hippocampal neurons survival and metabolic activity [73]. More recently, the secretome of HUCPVCs was also found to increase cell viability, proliferation, and neuronal cell densities in both cortical and cerebellar neuronal cultures [74]. Other* in vitro* and* in vivo* studies showed similar results [75–77].

MSCs application into SCI clinical trials has been widely studied and throughout them MSCs biosafety has been quite explored. In a Phase I/II clinical study, autologous BM-MSC transplantation as well as bone marrow stimulation with macrophage colony stimulating factor (GM-CSF) was used to treat complete SCI [78]. Likewise, the transplantation of* ex vivo* expanded autologous MSCs was also used in pilot clinical studies [79, 80]. Currently, autologous BM-MSCs implantation in an acute and chronic SCI at cervical and thoracic level is being used in a Phase I/II clinical trial [67]. Even though 90% of patients with acute cervical injuries showed significant improvement, only mild improvement was found in chronic patients. Nevertheless, a larger group of patients is needed to evaluate the efficacy of this therapy. Mononuclear BM cells transplantation for SCI treatment can also be used in alternative to BM-MSCs, as it was shown to have a similar efficiency* in vivo* [81]. In fact, the clinical safety and primary efficacy data of autologous BM-derived mononuclear cells for SCI were already studied in a Phase I/II clinical trial involving traumatic paraplegia (*n* = 215), traumatic quadriplegia (*n* = 49), and nontraumatic spinal cord myelopathy (*n* = 33) [82]. In this study, the cells were delivered through a lumbar puncture and a 3-month periodic follow-up study was designed to analyze neurologic and motor improvements, as well as safety parameters such as the therapeutic time window, CD34+ cell count, and influence of sex and age. At the end of the study, neurological status improvement was observed in one-third of SCI patients. Moreover, the outcome of the therapy was only influenced by: (1) the time elapsed between injury and treatment; and (2) the number of CD34+ cells that was injected [82].

According to all of these findings, MSCs may be equally powerful tools for SCI regeneration-based strategies.

### 2.5. Glial Cells

The possible role of other mature cells on the SCI regenerative process has attracted the attention of investigators in the field. For that purpose, glial cells including* olfactory ensheathing cells* (OECs) and* Schwann cells* (SCs) have been explored over the past decade.

#### 2.5.1. Olfactory Ensheathing Cells

OECs are glial cells that ensheath olfactory axons, within both the PNS and CNS portions of the primary olfactory pathway [83], and that are responsible for the successful regeneration of olfactory axons throughout the life of adult mammals [84]. These cells have a highly malleable phenotype, most likely due to coexpressing phenotypic features of astrocytes and SCs [85]. According to this theory, it is believed they can either switch from one type to another depending on their needs, or combine the roles of both when transplanted into an injury [83, 85].

At a glance, OECs might seem a curious choice for cell transplantation. The mammalian olfactory system is unique in supporting axonal outgrowth from its peripheral neuronal cell bodies in the olfactory epithelium into the CNS olfactory bulb, throughout life [86]. Furthermore, the expression of SCs-specific phenotypic features by OECs led to the hypothesis that these cells facilitate the growth and the myelination of axons within the CNS of adult mammals. The initial study that inspired OECs transplantation into CNS was performed by Ramon-Cueto and Nieto-Sampedro [87]. OECs were grafted into the dorsal-root entry zone of the postdevelopmental CNS. The grafted cells were able to promote the regrowth of transected dorsal roots, which was interesting since this is a region where normally dorsal root regeneration does not occur [87]. After these findings, numerous studies have demonstrated the effectiveness of OECs in supporting nonolfactory CNS axons growth and remyelination. Evidences showing the ability of OECs to myelinate dorsal root ganglion (DRG) neurons* in vitro* were firstly provided by Doucette and Devon in an* in vitro* coculture system [83, 88]. The myelination of DRG neurites by these glial cells was clearly observed and resembled the process by which SCs myelinate peripheral axons [83]. Subsequently, OECs were found to be able to remyelinate axons* in vivo *by Franklin et al. [89] and Imaizumi et al. [90]. In these studies, OECs were transplanted into an x-irradiated demyelinated area of the adult rat spinal cord. These cells remyelinated the existent axons after transplantation [89], which were found near and remote from the cell injection site, indicating extensive migration of OECs throughout the lesion [90]. Moreover, the remyelinated axons displayed improved conduction velocity and frequency-response properties, with action potentials being conducted at a greater distance into the lesion [90].

Although the effectiveness of OECs in supporting CNS regeneration was extensively studied and clearly showed, some negative reports have been presented. In a first line of evidence against this idea, Plant et al. [91] showed that OECs from adult rats did not myelinate DRG neurites. OECs failed to exhibit the so-described “Schwann-like” pattern of myelination. In contrast, “flat meandering processes” of OECs were observed encircling the DRG neurites [91]. Later on, the reparative ability of these cells in a contusion injury of the spinal cord was evaluated. After transplantation, OECs exerted a poor effect over axonal outgrowth and myelination, as well as functional hindlimb recovery of the animals [92].

As a conclusion, it is widely considered that OECs can create a permissive environment for axonal regeneration in the hostile environment of a SCI. While this is associated, by several authors, with the glial cell ability to support axonal growth and remyelination, others attribute this phenomenon to their secretome. In fact, OECs were found to secrete nerve growth factor (NGF), brain-derived neurotrophic factor (BDNF), neuregulins [31], and glial cell line-derived neurotrophic factor (GDNF) [32].

Regarding OECs transplantation into human SCI, some clinical trials have already been performed. The feasibility and safety of autologous OECs transplantation into patients with complete thoracic injuries was tested in a Phase I/II clinical trial [93, 94]. One year [93] and three years [94] after cell implantation into the damaged area, no complications were observed regarding the safety of the procedure. No spinal cord cyst or tumor formation was reported, neither neuropathic pain nor deterioration in neurological status. Also, there were no significant functional changes in any patients. In contrast, a Phase I/II pilot clinical study performed by Lima et al. [95] showed that the transplantation of olfactory mucosa autografts in patients with severe chronic SCI had promoted motor improvements in 11 patients (out of 20). Although some adverse events were reported in 5 of the patients, the growth of nonneoplastic tissue in the lesion site of all of them was observed.

#### 2.5.2. Schwann Cells

Over the years, it has been considered that SCs might be useful tools as cell therapies for CNS injuries such as SCI. This idea is based on the possibility that SCs might allow damaged CNS axons to regrow and remyelinate in the same way as it occurs in the PNS [96]. However, it has been postulated that the suitability of SCs could be diminished in the presence of astrocytes [97]. Recalling that this cell type is present in areas of SCI, such hypothesis would be imposing the idea that SCs transplantation within astrocyte-rich environment would unable these cells to integrate extensively within it [98]. Despite the evidences supporting this theory, there are several studies indicating that SCs are able to promote regeneration, while myelinating axons in SCI sites, thus being a good candidate to mediate the repair of such lesion. To corroborate this, experiments with autologous SCs transplantation were performed in thoracic injuries of cat spinal cords. In twelve animals (out of 25), all surviving axons in the dorsal column were remyelinated by the transplanted cells at injury level [99]. There was also a peripheral myelination of the dorsolateral tracts in six cases [99]. Furthermore, in a transected nude rat spinal cord, grafts of human SCs promoted axonal regeneration and myelination of several neuronal populations in the lesion site. Some regenerative growth also occurred beyond the graft, accompanied by a modest improvement in function [100]. More recently, adult SCs were found to sustain neuronal survival and promote axonal regeneration and hindlimb locomotor performance in a moderately contused adult rat thoracic spinal cord [92]. Thereafter, autologous transplantation of mitogen-expanded SCs in a model of acute demyelination of a monkey spinal cord resulted in functional and anatomical repair of the lesion, as well as in repair of large areas of demyelination [101].

Another interesting fact was that genetic-modified SCs that overexpress NGF [102] or BDNF [103] robustly increased axonal growth and remyelination after transplantation into SCI adult rats [102, 103]. Interestingly, grafted SCs exhibited a phenotypic and temporal course of differentiation that matched patterns normally observed after peripheral nerve injury [102].

So far, the evidences show the potential of SCs as cell transplants to integrate into SCI. As a result, their clinical translation has been described in a number of interesting reports. For instance, Saberi's group focused on the autologous transplantation of SCs into patients with chronic spinal cord injuries. The cells were injected directly into the lesioned area [104] or by intramedullary delivery [105]. In both studies, no adverse effects were observed one year [104] and two years [105] after cell transplantation, even though beneficial effects were not observed. In general, the procedures conducted were found to be safe. More recently, the Miami Project to Cure Paralysis performed the first-ever FDA approved SCs transplantation in a patient with complete thoracic SCI. The aim of this Phase I clinical trial is to evaluate the safety and feasibility of transplanting the patient's own SCs. Therefore, the patient received his own SCs about four weeks after injury and there have been no adverse consequences, so far. The project is now moving forward with this Phase I clinical trial, enrolling a total of eight participants with acute thoracic SCI [106].

Regardless of the advances in cell therapy for SCI treatment revealing to be promising, this approach is usually applied acutely and subacutely. However, cell transplantation for SCI often fails to yield functional recovery [13]. When cells are simply directly delivered into the injury site at this phase, an elevated percentage does not survive to the profound hypoxic and ischemic environment. Therefore, alternatives are needed in order to efficiently deliver cells and cell based therapies within SCI sites.

## 3. Biomaterials as a Tissue Engineering Approach for SCI Repair

The limited regenerative capacity of the CNS is well known. Besides the inhibitory environment that is created after damage, as it occurs in SCI, there is a lack of a physical matrix where neurons and endogenous repairing cells can adhere. These are two of the main reasons supporting the use of biomaterials in SCI-related research. In this sense, biomaterials science and tissue engineering approaches have been in the forefront of new strategies to approach SCI treatment. Among the biomaterials available, hydrogels appear as an excellent option, mainly due to their physical properties, which can closely mimic the soft tissues environment and the architecture of the CNS. Also, their chemical composition can be adapted to integrate extracellular matrix (ECM) molecules as well as other adhesion proteins, aiming at efficiently support and guide axonal regeneration. Interestingly, the development of hybrid matrices is also an approach used for SCI repair, since one can benefit from the properties of different materials to promote SCI recovery [107–109].

Taking this into account, this section will focus mainly on biomaterials application in a SCI context, particularly the use of hydrogel-based strategies.

### 3.1. Hydrogel-Based Biomaterials for SCI Treatment

For clinical applications, the design of a biomaterial must satisfy some essential criteria, such as biocompatibility, so it does not trigger any immune response from the host; specific tailored mechanical and physicochemical properties that allow both spinal cord stabilization and cell attachment and growth; porosity and permeability for the diffusion of ions, nutrients, and waste products; and biodegradability, so the biomaterial degrades as new tissue grows, thus mimicking the natural mechanisms of breakdown and synthesis of ECM in the natural tissues [14, 110, 111]. Among the variety of available materials for tissue engineering, hydrogels are particularly appealing for neural tissue repair, because their properties match all these requirements. Actually, hydrogels have physical properties that allow them to be injected into the body in a noninvasive manner. Moreover, they can be administered in a localized manner and are also able to fill the defects caused by injury [14, 112, 113]. Therefore, they act as depots for a sustained release of cells and molecules at the injury site. As cell delivery agents, hydrogels also improve cell survival and integration [114]. Structurally, they are very similar to macromolecular-based components in the body and are considered biocompatible, namely, when derived from natural polymers [115]. Also, their high water content has the advantage over other matrices of better mimicking the aqueous environment of the ECM [116].

A number of hydrogels have been developed for SCI repair, including natural-based hydrogels such as alginate [108, 117, 118], agarose [119–121], collagen [122–124], fibronectin [125, 126], fibrin [127, 128], matrigel [122, 129], and gellan-gum [109, 130, 131], as well as synthetic biodegradable-based hydrogels, namely, poly(lactic acid) (PLA) [132, 133], poly(lactic-co-glycolic acid) (PLGA) [134, 135], poly(ethylene glycol) (PEG) [136, 137], and the nonbiodegradable methacrylate-based hydrogels, including the poly(2-hydroxyethyl methacrylate) (pHEMA) [107, 122, 138] and poly(hydroxypropyl methacrylate) (pHPMA) [139–141].

### 3.2. Natural-Based Hydrogels

An important aspect to be considered when developing a hydrogel is its integration and interaction with the host tissue. Therefore, many of the hydrogel formulations used in biomedical applications include natural polymers or molecules present in living tissues.

For neural tissue repair, natural-based hydrogels are substances that normally appear in natural ECM or have certain properties that are recognized by cells, facilitating their integration within the host [142, 143], thus being preferred for SCI repair. Moreover, they exhibit similar properties of the soft tissues they are replacing [143]. However, since these materials derive from natural sources, they may elicit immune reactions from the host where they will be implanted and heterogeneity between batches may also be observed [144].

Among the above referred natural hydrogels, we will herein focus on agarose, alginate, collagen, fibrin, chitosan, and gellan-gum.

#### 3.2.1. Agarose

Agarose is a polysaccharide of D-galactose and 3,6-anhydro-L-galactopyranose that has tissue-like mechanical properties and has been widely used for drug delivery strategies due to its porous nature [120]. In addition, agarose gels have also the potential to be applied as nonviral gene delivery systems as they have been shown to provide a slow release of bioactive, compacted DNA [145]. Being derived from cell walls of red algae, agarose is a biocompatible component, which enables it to be used in tissue engineering approaches.

One aspect of agarose gels that makes them particularly interesting for CNS-related diseases is their ability to polymerize* in situ*, so they can fill different types of neurological defects, adapting to the shape of the lesion [120]. Moreover, this type of hydrogel has already shown the capacity of supporting neurite extension* in vivo *[120].

In two different rat models of SCI (contusion and dorsal-over hemisection), agarose gels were used as reservoirs for MP-loaded nanoparticles [146, 147]. This kind of construct allowed for a local and gradual release of the drug, with improved effects on reduction of the lesion volume and expression of proinflammatory proteins, when compared to systemic MP delivery [146, 147]. Agarose-based hydrogel has also been used for harboring lipid microtubes loaded with different drugs, namely, chondroitinase ABC (chABC) [148]. This system facilitates a local sustained release of chABC, consequently reducing the deposition of chondroitin sulfate proteoglycans (CSPGs, a major class of axonal growth inhibitors) and obviating the use of more invasive, continuous drug delivery systems (such as pumps or catheters) [148]. In an identical approach, agarose gels were coupled with lipid microtubes loaded with constitutively active Rho GTPases (Cdc42 and Rac1), which reduced CSPGs deposition and reactive astrocytes, promoting axonal growth in CSPG-rich regions [149]. More recently, a bioengineered agarose scaffold proved to support motor axon regeneration after a complete transection SCI model [150]. Moreover, the fabrication of channels within the gel allowed a more linear and organized axonal growth [121, 150]. In another study, agarose gels were modified to become photolabile and then, after the exposure to a focused laser, physical and chemical channels were created, by simultaneously immobilizing a fibronectin peptide of glycine-arginine-glycine-aspartic acid-serine (GRGDS) into their structure. These channels were found to provide guidance in cell migration and neurite outgrowth [151].

#### 3.2.2. Alginate

Another polysaccharide derived from cell walls of algae (brown algae) is alginate, which is able to absorb 200–300 times its own weight in water [152]. Composed of repeating units of (1–4)-linked *β*-D-mannuronate and *α*-L-guluronate [153], it has been used as a substrate for cell encapsulation, cell transplantation, and tissue engineering applications [108, 154, 155]. The gelation of this hydrogel occurs upon interactions between the carboxylic acid moieties and different counterions, like calcium [156]. However, the gelation procedure can be also based on the existence of a physical network, stabilized by intermolecular hydrophobic interactions between alkyl chains linked to the alginate backbone [154].

Alginate gels with hydrophobic domains provide a good retention of proteins that could be released upon the dissociation of the hydrophobic junctions [154].

In* in vivo* models, alginate hydrogels were also applied for the delivery of growth factors, including vascular endothelial growth factor (VEGF). After the application of a mechanical stress to the hydrogel, increased amounts of VEGF were released from the gels, leading to enhanced neovascularization processes within alginate hydrogels [157]. In acute cervical spinal cord lesions of adult rats, alginate-based highly anisotropic capillary hydrogels induced directed axon regeneration across the implanted artificial scaffold [108]. Since mammals do not possess enzymes capable of degrading high molecular polymers of alginate, the addition of PLGA microspheres loaded with alginate lyases to the gel can provide a tunable and controlled enzymatic degradation of this natural hydrogel [158]. In a more recent study, alginate hydrogels were used as deposits of GDNF (either free or inside microspheres) and injected into an injury of a hemisection model of SCI in rats. After either six weeks or three months, more neurofilaments were observed in the lesion of the animals treated with free GDNF loaded hydrogels, as compared to microspheres-GDNF-treated or untreated controls. In addition, the same group of animals presented less glial fibrillary acidic protein (GFAP) staining and more endothelial and nerve fiber infiltration at the lesion site. Superior functional recovery was also observed in free GDNF-treated rats, as assessed by gait analysis [118].

#### 3.2.3. Collagen

Collagen is one of the major proteins found in the ECM of different tissues in mammals [159]. It is mainly synthesized by fibroblasts and there are up to 29 different collagen types, the type I being the most common [159]. In addition, gel formation can be induced just by changing the pH of a collagen solution [143]. Collagen-derived materials are therefore highly biocompatible, but also biodegradable and noncytotoxic, having the ability to support cellular growth [159]. In this sense, collagen has been widely used in clinics, in different applications such as recovery of tissue defects, burns, wound dressings, and nerve regeneration [160]. As major drawbacks, collagen mechanical behavior* in vivo* may be variable and sometimes it may elicit an antigenic response, namely, if cross-species transplantation is used [161]. Other concerns include variability in the enzymatic degradation rate, when compared with hydrolytic degradation, and presence of trace impurities [159].

In what concerns collagen application to SCI, Jimenez Hamann et al. [162] developed a concentrated collagen solution for the localized delivery of different growth factors. Collagen with epidermal growth factor (EGF) and fibroblast growth factor 2 (FGF-2) was injected into the subarachnoid space of injured Sprague-Dawley rats. This resulted in less cavitation at the lesion epicenter (and also in other caudal areas), associated with more white matter sparing, as compared to nontreated animals [162]. In another study, collagen filaments were grafted parallel to the spinal cord axis of SCI rats, working as a bridge to foster neuronal regeneration. After four weeks, regenerated axons crossed the proximal and distal spinal cord-implant interfaces. Following twelve weeks, rats presented improved locomotor behavior and somatosensory evoked potentials (SSEP) were observed [123]. More recently, multichannel collagen conduits were used as reservoirs for neurotrophin-3 (NT-3) gene delivery in SCI rats. One month after injury, an aligned axonal regeneration was observed, and a higher number of regenerating axons were found in the conduits delivering NT-3 [124]. The association of collagen scaffolds to basic FGF also induced significant improvements in motor behavior of SCI rats and allowed guided growth of fibers through the implants [163].

#### 3.2.4. Fibrin

Hydrogels based on fibrin have also been extensively explored for SCI treatment. Fibrin is a fibrous protein that is involved in blood clotting. It is produced during the coagulation cascade, when fibrinogen is cleaved by thrombin, giving origin to fibrin monomers. Thereafter, these monomers spontaneously polymerize and create a three-dimensional (3D) matrix [164]. One important aspect of fibrin is the possibility to control their gelation process by varying the concentration of thrombin used. This feature offers the possibility of maintaining fibrin at a liquid state during injection, while forming a solid scaffold* in vivo* [165]. However, there are also some disadvantages. Fibrin gels from mammalian origin tend to degrade rapidly [166, 167] and may be easily contaminated by blood-derived pathogens or prion proteins [168]. In addition, some reports show that autologous mammalian fibrinogen inhibits neurite outgrowth [169] and activates resident astrocyte scar formation [170].

Regarding the use of fibrin in SCI applications, Iwaya et al. showed in 1999 that it was an effective intermediate for intraspinal delivery of neurotrophic factors [171]. In the same line of thought, Taylor et al. managed to deliver NT-3 within fibrin scaffolds to SCI rats. Nine days after injury, this treatment elicited a more robust neuronal fiber growth into the lesion, in comparison to control groups. A dramatic reduction of glial scar formation was also observed. However, no differences in motor recovery were found between groups [128]. More recently, with the purpose of avoiding some of the mammalian fibrin side effects, Sharp et al. tested salmon-derived fibrin as an injectable scaffold for SCI [165]. Salmon fibrin-treated animals showed greater recovery of locomotor and bladder function and even more serotonergic innervation caudal to the lesion, as compared to animals treated with human fibrin or untreated controls. Furthermore, no effects were observed on glial scar formation or lesion volume [165]. Additionally, in 2010 King et al. used injectable forms of fibrin mixed with fibronectin (FN/FB) to support axonal ingrowth after SCI [126]. One week after injury, the mixture showed good integration with the host spinal cord and supported some degree of axonal growth. After four weeks, axonal growth in FN/FB implants was the greatest compared to other implants tested [126].

#### 3.2.5. Chitosan

The linear polysaccharide chitosan is also a good alternative as a regenerative biomaterial-based strategy for SCI. This polysaccharide is composed of randomly distributed *β*-(1–4) linked D-glucosamine (deacetylated unit) and N-acetyl-D-glucosamine (acetylated unit). It can be derived from chitin found in crustacean shells, which is the second most abundant biopolymer after cellulose [172].

Chitosan is able to form a gel by itself, without the need of additives [173]. That may happen via hydrogen bonds, hydrophobic interactions, and chitosan crystallites [174]. These hydrogels can also be formed by blending chitosan with other water-soluble nonionic polymers [175] or polyol salts [176]. Since it is of polycationic nature in acidic conditions, chitosan can also form hydrogels through interaction with negatively charged molecules [177]. Another type of chitosan hydrogels can be formed via covalent bonds with metal ions [178], though these gels are less suitable for biomedical use [173]. Finally, the gelation of chitosan could also be obtained through covalent bonding between polymer chains. These bonds make the hydrogel more stable because the gelation is irreversible. Nevertheless, this approach may alter the primary structure of chitosan, which will lead to changes in its properties [173].

Chitosan hydrogels are pH-sensitive, being soluble in dilute aqueous conditions and precipitating into a gel at neutral pH [179]. The fact that this polymer is biodegradable and biocompatible is also very important for being used as a scaffold in tissue engineering applications. In vertebrates it is mainly degraded by lysozyme and some bacterial enzymes in the colon [180].

In what concerns neuronal repair, chitosan is commonly applied in the production of tubular structures most frequently used in peripheral nervous system [181]. However, chitosan hydrogels have also been applied in neural tissue engineering. For instance, the use of chitosan/glycerophosphate salt (GP) hydrogels showed that this type of gels provides a suitable 3D scaffolding environment for neurons, namely, fetal cortical mouse cells [179]. Addition of peptides, like poly-D-lysine, also showed the capacity to improve scaffold biocompatibility and nerve cell affinity for chitosan materials [182].

#### 3.2.6. Gellan-Gum

Finally, the recent use of gellan-gum- (GG-) based hydrogels for CNS applications has already been shown to be promising. GG is a natural polysaccharide that is produced by the bacterium* Pseudomonas elodea* [183]. Its structure consists of repeating units of a tetrasaccharide, composed by two residues of D-glucose, one residue of L-rhamnose and another of D-glucuronic acid [D-Glc(*β*1→4)D-GlcA(*β*1→4)D-Glc(*β*1→4)L-Rha(*α*1→3)]n [184]. This linear anionic polysaccharide exists in both the acetylated and deacetylated forms, originating thermoreversible gels with different mechanical properties according to the degree of deacetylation [183].

GG is noncytotoxic and particularly resistant to heat and acid stress, being useful in culture of extremophile organisms [185]. The gelation process of this biomaterial is ionotropic, meaning that the presence of cations is necessary for the formation of a stable hydrogel structure [186]. In this process, divalent cations promote a more efficient gelation than monovalent cations [187], at the same time that several structural changes take place. At higher temperatures, GG is in a coil form. As temperature decreases, there is a thermoreversible transition from coil to double-helix structures. These structures form oriented bundles by self-assembly, which are called junction zones. Untwined regions of polysaccharide chains can also link with the junction zones, leading to the formation of a three-dimensional network that assembles the gel [187].

Regarding SCI applications, our group has developed different strategies based on GG hydrogels [109, 131]. In 2010, Silva et al. [109] conjugated GG with three-dimensional tubular structures made of a biodegradable blend of starch (SPCL). This construct was revealed to be noncytotoxic and capable of supporting the* in vitro* culture of oligodendrocyte-like cells. Moreover, when applied* in vivo* in a hemisection rat SCI model, it was shown that the scaffold was well integrated in the lesion site without eliciting any chronic inflammatory processes [109]. In 2012, the same construct was adapted to enhance osteointegration by premineralizing the external surfaces of the SPCL structure [131]. By using a sodium silicate gel as nucleating agent, it was possible to create two distinct environments, one aimed at inducing osteogenic activity (external surface) and another for fostering neuroregeneration (internal surface) [131].

A common modification employed in this type of hydrogels is the addition of different peptide sequences that mimic the ECM [151, 188], with the purpose of improving phenomena like cell adhesion, growth, and development [189]. In this sense, our group has modified GG with GRGDS fibronectin peptide, which resulted in the enhancement of cell proliferation and metabolic activity, as will be described in detail in the next section [130, 190].

### 3.3. Synthetic Hydrogels

Regarding synthetic hydrogels, their biggest advantage is the fact that they can be tailored to fit the needs for a certain application. From physical and chemical properties to degradation rates, many aspects of their structure can be modulated in order to improve their biocompatibility and degradation rate [191]. The findings related with the use of biodegradable poly(lactic acid) (PLA) and poly(lactic-co-glycolic acid) (PLGA) hydrogels, methacrylate-based hydrogels, and poly(ethylene glycol) (PEG) hydrogels will be briefly discussed here.

#### 3.3.1. Poly(lactic acid) (PLA) and Poly(lactic-co-glycolic acid) (PLGA)

PLGA/PLA polymers are members of the *α*-hydroxy acid class of compounds and are composed of synthetic biodegradable aliphatic polyesters [192]. For controlling the degradation rate and mechanical properties of these polymers, it is possible to vary the ratio of monomer units and their stereochemistry (either D- or L-form), as well as the molecular weight distribution of their chains [193]. Since PLGA and other similar polymers have been approved by the FDA for use in the repair of human peripheral nerves, their translation into CNS-related injuries seems promising [194].

In SCI applications, Patist et al. [138] tested the effects of poly(D,L-lactic acid) macroporous guidance scaffolds (in the form of foams), with or without BDNF, on a model of transected rat spinal cord. Foams were embedded in fibrin glue containing acidic-FGF, resulting in some gliotic and inflammatory response in the cord-implant interfaces. In addition, in BDNF-containing foams, 20% more NeuN-positive cells (marker for neurons) were present in the spinal nervous tissue in the rostral stump, as compared to controls, four and eight weeks after implantation, respectively. These same foams showed a significant higher level of vascularization. Curiously, treatment with fibrin only yielded more axons than the other groups. Through behavioral analysis, similar functional improvements in all groups were found [138]. Furthermore, PLA microfibers, in an aligned or random form, were implanted in rats subjected to a complete transection of the spinal cord. Four weeks after injury, both types of microfibers facilitated the infiltration of host tissue and allowed the closure of the initial three millimeters gap. However, aligned PLA fibers promoted longer distance of rostrocaudal axonal regeneration as compared to random PLA fibers or film controls [133].

Regarding PLGA, nano- and microparticles of this hydrogel have been widely used as delivery agents for tissue engineering applications [195]. In a SCI animal model, Fan et al. [135] used PLGA nerve conduits in combination with recombinant human NT-3 (rhNT-3). Rats were subjected to a complete thoracic transection of the spinal cord and then PLGA was implanted together with an rhNT-3 single dose administration. Animals treated with the combinatorial approach presented significantly improved performances in the BBB^2^ (Basso, Beattie, and Bresnahan) rating locomotor scale and grid walk tests [135].

#### 3.3.2. Methacrylate-Based Hydrogels

*Poly[N-2-(hydroxypropyl) methacrylamide] (PHPMA).* PHPMA hydrogels were first described by Woerly and colleagues [140, 141]. They synthesized a biocompatible and heterogeneous hydrogel, with an open porous structure that allowed the transport of both small and large molecules, as well as the migration of cells and blood vessels [141]. This hydrogel also presented viscoelastic properties similar to the neural tissue [140]. When implanted into a transected rat spinal cord, the hydrogel successfully bridged the tissue defect favoring cell growth, angiogenesis, and axonal growth within the microstructure of the network [140]. This hydrogel was showed to be permissive to the growth of a reparative tissue, composed of glial cells, blood vessels, axons, and dendrites and even ECM molecules, such as laminin and/or collagen [141]. Other features of PHPMA hydrogels include a reduction of necrosis and cavitation in the adjacent white and gray matter of transected rat spinal cords [139]. Furthermore, using this type of hydrogels in cats subjected to a transection lesion provided some motor benefits, as compared to nontreated cats [196]. More recently, PHPMA hydrogels were used as a matrix in order to create an appropriate microenvironment for axonal regeneration in SCI rats. Hydrogel-implanted animals exhibited an improved locomotor BBB^2^ score and an overall better coordination in neuromuscular evaluations, such as breathing adjustment to electrically evoked isometric contractions and H-reflex recovery. After immunohistochemistry analysis, ED-1 positive cells accumulation (macrophages/monocytes) was evident at the border of the lesion. At the same time, a larger number of neurofilament-H positive axons penetrated the matrix. In addition, there was also myelin preservation rostrally and caudally to the lesion [197].

*Poly(2-hydroxyethyl methacrylate and 2-hydroxyethyl methacrylate-co-methyl methacrylate) (PHEMA/PHEMA-MMA).* As other synthetic hydrogels, PHEMA/PHEMA-MMA polymers have the disadvantage of being nonbiodegradable [107, 122]. Nevertheless, this property allows them to remain stable, even upon implantation [144]. In addition, these are biocompatible hydrogels, with the capacity of swelling in water and retaining significant amounts of water without dissolving [198].

PHEMA polymers are the most actively researched nondegradable materials used for nerve guidance channels [193], because they possess soft, tunable mechanical properties and can be easily molded into tubular shapes, with controlled dimensions, morphology, and permeability [199]. Furthermore, since PHEMA synthesis is carried out at low temperatures and without toxic solvents, it is possible to incorporate bioactive compounds into the polymer scaffold [107].

When applied in a rat transection model, PHEMA-MMA hydrogel conduits allowed a continuity of tissue within the synthetic guidance channels created [107]. These conduits were further combined with different matrices and growth factors, leading to increased axonal density within the channels, as compared to unfilled channel controls [122]. Nevertheless, it was shown that the degree of integrity of the conduits was drastically reduced 16 weeks after implantation, when compared to eight weeks' time point [200]. Moreover, an important improvement was performed on PHEMA conduits by introducing coils into nerve channel's walls in order to provide reinforcement [201]. For instance, PHEMA-MMA guidance channels containing poly-caprolactone coils showed greater patency (openness) than nonreinforced channels, resulting in regeneration similar to autografts, regarding peripheral nervous system injury [201]. PHEMA sponges have also been used as a conductive substrate for regenerating axons in rats subjected to a spinal cord contusion lesion. These sponges were impregnated with collagen prior to implantation into the dorsal* funiculus* after the lesion. Two and four months after implantation, a minimal fibroglial reaction was observed, associated with low accumulation of mononuclear cells or angiogenesis within the sponge and spinal cord interface. Moreover, the cystic cavity was virtually absent and axons labeled with anterograde tracers penetrated and elongated through the full length of the sponge [202]. Modified PHEMA-based hydrogels have also been used in order to increase cellular adhesion [203]. For instance, a hydrogel structure modification with laminin-derived peptides—tyrosine-isoleucine-glycine-serine-arginine and isoleucine-lysine-valine-alanine-valine (YIGSR and IKVAV)—led to a significant increase of DRG cells survival, after two days in culture, as compared to unmodified hydrogels [203]. Furthermore, implanted PHEMA hydrogels in a model of partial cervical hemisection injury in rats have only induced a modest cellular inflammatory response, which disappeared after four weeks. In addition, minimal scarring was observed around the matrix. A considerable level of angiogenesis was observed within the hydrogels and, when soaked in BDNF, axonal penetration into the gel was observed [204]. In another model of complete transection of the cord, PHEMA hydrogels were implanted either immediately or one week after SCI. Three months later, histological evaluation revealed that the hydrogel adhered well to the spinal cord tissue. In addition, an ingrowth of connective tissue elements, blood vessels, neurofilaments, and Schwann cells throughout the gel was observed. Moreover, there was a significant reduction in pseudocyst volume, which was more evident in animals treated one week after injury [205]. More recently, Kubinová et al. used PHEMA hydrogels with oriented pores and modified with SIKVAV peptide in a spinal cord hemisection model. From three types of hydrogel tested (with different elastic modulus and porosities), the best option promoted tissue bridging and an aligned axonal ingrowth [206].

#### 3.3.3. Poly(ethylene glycol) (PEG)

Poly(ethylene glycol) (PEG) is a nontoxic polyether compound that is water soluble and known to resist protein adsorption and cell adhesion [207]. These properties make PEG polymer highly resistant to recognition by the immune system after implantation [144]. Besides this, PEG helps to seal cell membranes after injury, limiting cell death [144].

Depending on the cross-links created, PEG hydrogels can be designed with varying degradation rates and can be used as drug releasing vehicles [208, 209]. Moreover, they can be additionally modified in order to increase cell adhesion [210, 211]. It is also known that PEG exhibits rapid clearance rates and has already been approved for a wide range of biomedical applications [208], including SCI.

In an* in vivo *model of SCI, treatment with a PEG solution by itself was capable of accelerating and enhancing the membrane resealing process, restoring neuronal membrane integrity. This led to suppressed levels of reactive oxygen species (ROS) elevation and lipid peroxidation [136]. In a similar approach, PEG treatment was also able to restore the conduction of compound action potential (CAP) in injured spinal cords [137]. Furthermore, a study performed on adult guinea pigs showed that, six hours after a spinal cord contusion, a single subcutaneous injection of PEG (in saline) produced a rapid recovery of SSEP propagation through the lesion. This was followed by a significant recovery of the cutaneous* trunci* muscle (CTM) reflex, which is a good index of white matter integrity [212]. In another study, using dogs as an animal model of SCI, PEG injection in the acute phase was shown as clinically safe and induced a rapid recovery in different outcome measures, as compared to conventionally treated dogs [213]. Also, coupling PEG hydrogels with NT-3 and implanting these in a rat model of SCI provided improved locomotor behavior to lesioned animals and greater axonal growth, in comparison to controls treated with hydrogel alone [214]. More recently, it was also shown that PEG was effective even in conditions of low Ca^2+^ and low temperature and that the hydrogel mechanism of action may be based on a reduction of membrane tension, facilitating the resealing of the membrane [215].

In conclusion, it seems that PEG action has two main pathways: one is based on the protection against membrane damage, which leads to reduced necrosis and apoptosis, while the other is preventing the effects of mitochondria-derived oxidative stress, showing a reduction in ROS formation and lipid peroxidation [216].

### 3.4. Self-Assembly Peptides

Another alternative that has been used in SCI research is the application of self-assembling peptides (SAPs) [217]. These SAPs originate solid scaffolds that are formed by self-assembly of peptide amphiphiles from aqueous solutions [217]. The peptide sequences can be customized for obtaining a specific cell response. When cell suspensions are added to these aqueous solutions, the amphiphilic molecules aggregate forming different nanofiber networks. This aggregation happens mainly due to (1) the presence of electrostatic repulsions between the negatively charged SAPs and the cations present in culture media; and (2) the partial hydrophobic nature of the SAPs. An injection of liquid SAPs into living tissues will also lead to scaffold formation [217]. The presence of a peptide of interest in the hydrophilic part of the SAPs allows a significant motif presentation to cells. Based on this concept, in 2004, Silva and coworkers developed a SAP with IKVAV laminin motifs [217]. Neural progenitor cells (NPCs) were encapsulated in these gel-like scaffolds and remained viable for at least 22 days. Furthermore, this system was able to promote NPCs migration and direct their differentiation largely into neurons, while suppressing astrocyte differentiation [217]. This was proved to be due to IKVAV presence, since a similar SAP designed with the nonbioactive EQS (glutamic acid, glutamine, and serine) peptide did not induce cell migration, sprouting of neurites, or neuronal differentiation [217]. Suppressing astrocyte differentiation and proliferation is important since it can be associated with prevention of glial scar formation [217]. Finally, neurons within these networks were larger and produced longer neurites compared to neurons grown in control cultures. Later in 2008, a work published by the same group assessed the effects of SAPs with IKVAV motifs in a mouse compression model of SCI [218]. Twenty-four hours after injury, SCI mice were treated with a single injection of the IKVAV peptide amphiphiles (IKVAV-PA) and the respective controls. First, IKVAV-PA was found to be stable, being only biodegraded after 4 weeks. Then, the* in vivo* application of these peptide amphiphiles to SCI mice reduced the progression of astrogliosis (assessed after 5 and 11 weeks) and cell death (less apoptotic cells after 10 days). At the same time, there was an increased number of oligodendroglia at the site of injury, as compared to controls. The IKVAV-PA also promoted the regeneration of both descending motor and ascending sensory fibers through the lesion site 11 weeks following injury, even though fibers grew in a random manner. In addition, mice treated with IKVAV-PA presented a significant behavioral improvement as assessed by the BBB^2^ locomotor scale [218]. An injection of the IKVAV peptide alone did not induce functional recovery, which reinforces the idea that the combination of SAPs and IKVAV peptide is essential to produce an effect. In another study of the same authors [219], an injection of IKVAV-PA provided functional improvements both in mice and rats and in two different models of SCI (compression and contusion, resp.). Moreover, the IKVAV-PA treated animals presented a significantly higher density of serotonergic fibers, caudal to the injury site. Interestingly, this difference only appeared in the chronically injured cord [219]. The improved serotonergic innervation may partially explain the functional improvements observed in other studies [219].

More recently, Berns et al. [220] used a similar strategy by modifying these aligned scaffolds with IKVAV or RGDS epitopes. These ECM-derived bioactive peptides were presented on the surface of aligned nanofibers of the monodomain gel. The growth of neurites from neurons encapsulated within the scaffolds was enhanced, while the alignment guided the neurites along the direction of the nanofibers [220]. This fiber alignment proved to be a powerful directional cue for neurite outgrowth [220]. In addition, neurons cultured in the scaffold for two weeks presented spontaneous electrical activity and established synaptic connections [220]. Finally, when applied* in vivo*, the scaffolds were able to form* in situ* within the spinal cord and promoted the growth of oriented processes [220]. In summary, this particular scaffold has the potential to propagate electrical signals and neurite outgrowth in a desired direction [220].

Using a different SAP, firstly introduced by Dong et al. [221], Liu et al. tested the effects of a multidomain peptide [glutamine, leucine, and lysine—K_2_(QL)_6_K_2_ or (QL6)] on a rat SCI compression model [222]. QL6 was injected 24 h after injury and led to a significant reduction in posttraumatic apoptosis, inflammation, and astrogliosis. In addition, it promoted tissue preservation and axonal regeneration. SCI rats treated with QL6 also presented significant motor recovery, as assessed by the BBB^2^ test [222].

Another interesting fact about SAPs is that, by modulating their mechanical properties, particularly their stiffness, it is possible to influence neuronal differentiation and maturation [223]. In line with this, Sur et al. [223] studied the morphological development of hippocampal neurons when using SAPs with different fiber rigidities. Softer nanofiber substrates provided an accelerated development of neuronal polarity and the weaker adhesion of neurites to soft PA facilitates easier retraction, which fosters the frequency of “extension-retraction” events [223].

According to the reported findings, hydrogels may have a high therapeutic value for SCI treatment. Therefore, their future application for cell and/or drug delivery appears to be promising. In addition, considering the previously reported limitations often found within cellular based therapies, the combination with biomaterials has been widely considered as an alternative method to mediate cellular transplantation more effectively.

## 4. Combining Biomaterials and Cell Transplantation for SCI Treatment

**Figure 2:**
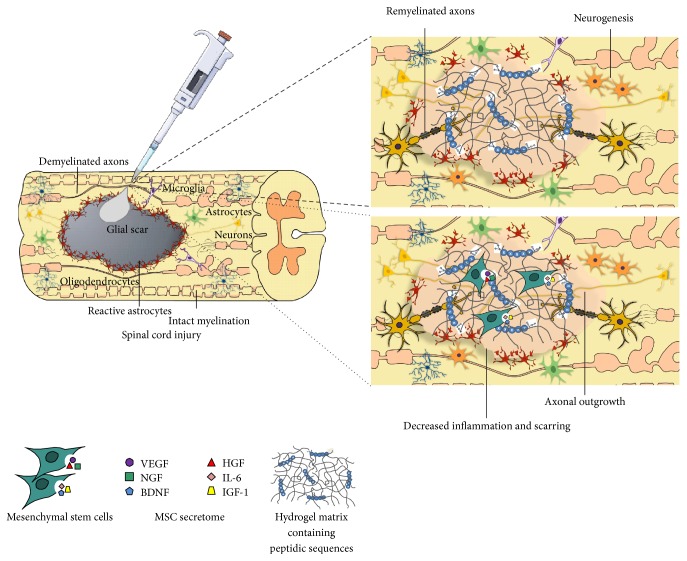
The use of hydrogel matrices or their combination with cell therapy, such as MSC transplantation, for SCI treatment might potentiate axonal regeneration and outgrowth through the injury site.

In spite of the experimental ground work regarding cell transplantation and biomaterial-based therapies for SCI, their use as single approaches presents some limitations. Regarding biomaterials alone, it is not always easy to modulate their properties so they respond exactly as expected. Moreover, they are not able to replace the cells lost during SCI. On the other hand, cell transplantation by itself is not capable of recreating spinal cord complex architecture and stability, or even direct axonal regrowth [11]. Hence, taking advantage of what both therapies offer to overcome the multiple hurdles of SCI, synergistic effects on regeneration and functional recovery of the injured spinal cord can be provided if combined strategies are employed [11, 14] (Figure 2).

In this sense, researchers have been focusing on the use of biomaterials, specifically hydrogels, as systems for cell encapsulation and delivery into injured spinal cords. As summarized in Table 1, the advantages of these combinatorial approaches have been revealed in several studies.

Regarding NSCs, artificial scaffolds made of synthetic poly(glycolic acid) (PGA), PLA, and their copolymers have been shown to be promising as cell carriers [224, 225]. However, the NSCs behavior was found to be dependent on the mechanical characteristics of the scaffold, as the rate of NSCs differentiation was higher in PLA nanofibers comparing to microfibers, independently of the alignment [225]. Furthermore, the transplantation of NSCs with PLGA scaffolds into SCI rats has been shown to maintain cell viability for longer periods of time and improve the functional recovery of the rats [226, 227]. In another interesting study, NSCs and endothelial cells (ECs) were codelivered in a two-component biomaterial composed of an outer PLGA scaffold and an inner poly(ethylene glycol)/poly-L-lysine (PEG/PLL) macroporous hydrogel to the injured rat spinal cord in a hemisection model of SCI. The role of ECs in this approach was to promote the vascularization of the scaffold in order to increase NSCs survival. In effect, the number of functional blood vessels at the lesion site has increased, though NSCs survival has not, compared to the implant carrying only cells [228]. The advantages of natural gels as a biomaterial to modulate NSCs were also revealed in several studies. For instance, alginate sponges contributed to the survival and differentiation of rat hippocampus-derived neurosphere cells, after transplantation into injured rat spinal cords [229]. Following a similar line, fibrin based hydrogels supported neurite outgrowth [230].* In vivo* they have also been shown to increase the number of neural fibers in a subacute rat model of SCI, delaying simultaneously the accumulation of GFAP positive reactive astrocytes around the lesion [231]. More recently, NSCs expressing GFP were embedded into growth-factor cocktail-containing fibrin matrices and were found to differentiate into neurons that were able to form synapses with the host cells. Moreover, specific signaling pathways were found to influence large axonal extension along the injury site. Animals' functional recovery was also observed [22]. After chitosan/chitin films were shown to promote cell survival* in vitro* [232], chitosan-based channels coated with laminin were shown to significantly improve spinal cord-derived NSCs survival, twelve weeks after transplantation in the injured rat spinal cord. Still, axonal regeneration as well as functional recovery was not promoted [233]. Finally, GG hydrogels were also used to engraft NSCs in an* in vitro* study performed by Silva et al. [130]. To enhance cell adhesion, GG hydrogels were modified with GRGDS peptide using Diels-Alder click chemistry. NSCs were found to adhere and proliferate within the modified gels, when compared to unmodified ones. In addition, OECs were used to further enhance NSCs survival and outgrowth in this system. In the cocultures, NSCs presented significantly greater survival and proliferation compared to monocultures of NSCs [130].

MSCs combination with biomaterials has also emerged as a potential tissue engineering approach, aiming at increasing both MSCs engraftment efficiency and survival at the injury site. For instance, therapeutic BM-MSCs in a poly(D,L-lactide-co-glycolide)/small intestinal submucosa (PLGA/SIS) scaffold induced nerve regeneration in a complete spinal cord transection model [234]. Different defect lengths were studied, with BM-MSCs survival being observed in general. In addition, axonal regeneration as well as functional recovery was also reported, though it was found to be dependent on the defect length—smaller defects allowed for higher functional recovery and regeneration [234]. Spinal cord regeneration has also been extensively studied regarding the implantation of MSC-containing macroporous hydrogels based on derivatives of 2-hydroxyethyl methacrylate (HEMA), 2-hydroxypropyl methacrylamide (HPMA), or copolymers of HPMA [235] into spinal cord injuries. These hydrogels were either modified by copolymerization with a hydrolytically degradable crosslinker (N,O-dimethacryloylhydroxylamine) or by different surface electric charges (HEMA-sodium methacrylate (MA) negative charge; HEMA-2[2-(methacryloyloxy)ethyl]trimethylammonium chloride (MOETACl) positive charge). After implantation, the hydrogels integrated well in the injury site and promoted cellular ingrowth, more pronounced in the positively charged HEMA/MOETACl hydrogels. Axons were found to invade all the implanted hydrogels from both proximal and distal stumps. Moreover, the hydrogels were resorbed by macrophages and replaced by newly formed tissue containing connective tissue elements, blood vessels, astrocytic processes, and neurofilaments. P(HEMA) and HPMA hydrogels were also modified with laminin-derived Ac-CGGASIKVAVS-OH peptide sequences [236] and RGD amino acid sequences [237], respectively. These significantly increased the number of attached cells and their growth area [236] and allowed the hydrogels to successfully bridge the spinal cord cavity, while promoting axons infiltration within it, as well as blood vessels and astrocytes growth [238].

Agarose, alginate, and matrigel are other natural hydrogels used for MSCs transplantation. Template agarose scaffolds were grafted with BM-MSCs expressing either NT-3 [121] or brain-derived neurotrophic factor (BDNF) [150] and placed into spinal cord lesion site. Long-tract sensory axonal regeneration with increased linear organization was observed into the spinal cord, even into severe, complete spinal cord transection sites [150]. However, the formation of a host reactive cell layer in the interface of the scaffold prevented axonal penetration [121]. Regarding alginate and matrigel, an* in vitro* study has reported the potential of these gels in promoting DRG axonal regeneration when grafted with different cell types, including BM-MSCs [239]. The incorporation of fibronectin in alginate was also considered. While alginate alone inhibited both cell proliferation and DRG neurite outgrowth, which was attenuated by the addition of fibronectin or BM-MSCs, matrigel stimulated both cell proliferation and DRG neurite outgrowth, in either the absence or presence of cells. Fibrin has also been used to transplant GFP-positive BM-MSCs into the cavity formed after a hemisection spinal cord injury model in the rat. Four weeks after transplantation, increased cell survival as well as migration throughout the hydrogel was observed, accompanied by functional improvement of the animal, in comparison to animals that received just BM-MSCs or a vehicle control of PBS [240]. More recently, GG was also suggested for MSCs encapsulation [190]. The engraftment of BM-MSCs within a GG hydrogel modified with GRGDS fibronectin-derived peptide as previously described [130] increased cell proliferation and metabolic activity, when compared to unmodified hydrogels. Moreover, BM-MSCs secretome was positively influenced, as proven by the enhancement of the survival and differentiation of primary cultures of hippocampal neurons* in vitro *[190].

Taking into account the well known capacity of OECs to support and guide axonal elongation [89] and also their interesting results when transplanted into SCI lesion models [241, 242], their combination with a 3D matrix also holds great promise regarding SCI repair. Among the various studies exploring the combination of biomaterials and OECs, Novikova et al. [239] used an* in vitro* model to test OECs biocompatibility (among other cells) with different hydrogels. In alginate hydrogels, OECs presented an atypical spherical shape and their metabolic activity was inhibited. However, when alginate was complemented with fibronectin, OECs were the only cells able to proliferate. When OECs were cultured in matrigel, their proliferation was stimulated and their typical morphology was preserved [155]. Another* in vitro* study explored the biocompatibility of IKVAV self-assembling peptide nanofiber scaffold hydrogels using OECs. Either on 2D or on 3D surfaces, OECs could survive and migrate in the scaffolds. In addition, cell number, viability, and morphology were not significantly different compared to OECs cultured with poly-L-lysine [243]. More recently, Chan et al. [244] tested OECs ability to grow on polyhydroxybutyrate-polyethylene glycol hybrid polymers (PHB-b-DEG). OECs proliferation was enhanced when cultured in PHB-b-DEG films. Moreover, no cytotoxic responses were observed, and cell viability was maintained. Finally, it was also shown that OECs grown in PHB-b-DEG films entered into the DNA replication (S) phase and mitotic (G2-M) phase during the cell growth cycle, being associated with low apoptosis [244]. Moving to* in vivo *experiments, Ferrero-Gutierrez et al. [245] assessed the locomotor recovery of SCI rats when treated with a serum-derived albumin scaffold seeded with both OECs and ASCs. First, it was shown that both cell types adhered to the scaffold, remained viable, and expressed specific markers. Then, rats treated with the cell-seeded scaffolds showed improved locomotor skills at different time points, when compared to untreated SCI animals. Furthermore, there was a reduction in glial scar formation and the presence of cells expressing markers of neurons and axons at the injury site was observed [245].

Finally, the use of biomaterials for SCs encapsulation has also been considered. Although these cells belong to the peripheral nervous system, SCs application in a SCI context is quite common [246, 247]. This is mainly due to SCs myelinating capacity [248]. Therefore, the conjugation of SCs with biomaterials-based strategies seems an obvious step towards SCI regeneration. In a work developed by Novikova et al. [239], SCs presented an atypical shape and an inhibited metabolic activity when they were cultured on alginate hydrogels. However, the combination of both attenuated alginate inhibitory effects over DRG neurites outgrowth. In addition, SCs proliferation was stimulated when cultured on matrigel [155]. In another work from the same authors, SCs were cultured on biodegradable tubular conduits made from poly-beta-hydroxybutyrate (PHB) [249]. Then, the scaffold was implanted in SCI rats and the presence of SCs allowed the infiltration of neurofilament-positive axons within the conduits, associated with numerous raphaespinal and calcitonin gene related peptide- (CGRP-) positive axons. Therefore, this conjugate seems to support axonal regeneration after SCI [249]. In fact, the association of SCs with guidance structures has been recurrent in SCI experimental approaches [129, 250]. For instance, Bamber et al. [129] tested a SCI graft, where cells were seeded on mini-guidance channels composed of 60 : 40 polyacrylonitrile : polyvinylchloride copolymer (PAN/PVC). This construct, associated with the delivery of neurotrophins, promoted axonal outgrowth from the mini-channels into the distal host spinal cord [129]. An identical approach was performed by Fouad et al. [250], where SCs were grafted in 60 : 40 PAN/PVC channels and transplanted into the site of injury of SCI rats. This was complemented with chABC delivery and OECs transplants. This combined therapy provided significant improvements in the BBB^2^ locomotor score, which was correlated with an increased number of myelinated axons in the SCs bridge [250]. Tubular scaffolds made of high-molecular-weight poly(L-lactic acid) (PLLA) mixed with 10% PLLA oligomers were also used to seed SCs and implanted on rats subjected to a complete transection of the spinal cord [251]. This construct was able to hold without collapsing four months after injury. Through several time points analyzed, SCs remained present in the tubes, which were quite vascularized. In addition, there were a significant number of myelinated axons. However, after two months the growth and myelination presented a slight decrease [251]. In another interesting approach, Kamada et al. [252] differentiated BM-MSCs into SCs* in vitro*. Then, BM-MSC-derived SCs (BM-MSC-SCs) together with matrigel were used to fill an ultra-filtration membrane tube. This construct was grafted into the gap of completely transected spinal cords of adult rats. In these animals, the number of neurofilament- and tyrosine hydroxylase- (TH-) immunoreactive nerve fibers was significantly higher when compared to control groups. In addition, the same animals showed a significant recovery of the hindlimb function [246]. More recently, the same group combined BM-MSC-SCs with matrigel. This mixture was injected into the lesion site, 9 days after a contusion lesion in adult rats. The results demonstrated that, in comparison to control groups, BM-MSC-SCs with matrigel-treated animals presented a smaller cystic cavity area, a higher number of growth associated protein-43 (GAP43) positive fibers, a larger number of TH- or serotonin-positive fibers at the lesion epicenter and at a caudal level, the formation of peripheral-type myelin near the lesion epicenter, and a significant recovery of hind limb function [246].

In a contusion injury model, Patel et al. [247] implanted SCs with* in situ* gelling laminin/collagen matrices. In comparison to cell transplantation by itself, the 3D matrices enhanced long-term cell survival, but not proliferation. In addition, graft vascularization was improved and the degree of axonal ingrowth was also increased. Finally, some level of functional recovery was also achieved, as assessed through the BBB^2^ locomotor score [247].

These are very promising results regarding the use of biomaterials as cell carriers for SCI treatment. In the future, the challenge will be to define the most promising biomaterial to engineer and design effective cell-based therapies.

## 5. Conclusions

The inability of the adult CNS to regenerate is not completely understood regarding the mechanisms that are responsible for repressing axonal regeneration and spinal cord functional recovery. However, extensive progress has been made in neural regeneration in SCI. Therefore, we herein focused on some the most promising therapies currently used for SCI repair: cell- and biomaterial-based therapies and their conjugation.

Accomplishing axonal regeneration and reconnection across the lesion is the major goal for SCI repair [2]. Clearly, the use of cell transplantation is one of the top promising strategies for this kind of treatment. Their translation to human clinical applications is currently ongoing, with issues regarding cell biosafety and biocompatibility being extensively tested. Nevertheless, the efficacy of cell therapy is still compromised by the innumerous barriers presented by SCI, including significant cell death observed following transplantation, which clearly decreases the effectiveness of this technique. Besides, in chronic SCI, cell transplantation is not sufficient to promote tissue remodeling and axonal regeneration across the dense glial scar. Thus, regenerative strategies using scaffolds to bridge the two segments of the injured spinal cord and provide a three-dimensional environment for the regenerating axons are very attractive. In line with this, the advantages of using biomaterials that support cell transplantation were highlighted. However, the evolution of sophisticated 3D scaffolds from 2D conditions for such microenvironment of SCI is not free of challenges [193]. From requirements such as oxygen availability and nutrients diffusion for the encapsulated cells, to the variability on gradients and defects that result in heterogeneities in the synthetic microenvironment, there are many aspects that must be considered for the culture of mammalian cells in 3D environments, since it is already established that cell survival and differentiation and tissue homeostasis are highly dependent on these conditions [253].

Nevertheless, with increasing knowledge on the mechanisms by which specifically designed biomaterials support cell behavior, and thus how CNS regeneration is promoted, the future of SCI regeneration is probably linked to combinatorial approaches, integrating the multiple stimuli from these two elements. Meanwhile, advanced studies on how biomaterials modulate cellular activity and the biosafety and efficacy of this therapy must be addressed, in view of its clinical application. In this way, medicine and tissue engineering must work together in order to create better therapies.

## Figures and Tables

**Table 1:** Therapeutic potential of combinatorial approaches based on cell therapy and biomaterials for SCI treatment.

Cells	Biomaterial	*In vitro* improvements	*In vivo *		References
			Functional improvements	Histological improvements	
NSCs	PLA	✓	—	—	[225]
	PLGA	—	✓	✓	[226, 227]
	Alginate	—	✓	✓	[229]
	Fibrin	✓	✓	✓	[230, 231]
	Chitosan	✓	✓	*✗*	[232, 233]
	Gellan-gum	✓	—	—	[130]

NSCs plus ECs	PLGA-PEG/PLL	—	*✗*	✓	[228]

NSCs plus OECs	Gellan-gum/GRGDS	✓	—	—	[130]

MSCs	PLGA/SIS	—	✓	✓	[234]
	HEMA, HPMA, and HPMA copolymers	—	✓	✓	[235, 236, 238]
	Agarose	—	*✗*	✓	[121, 150]
	Alginate	✓	—	—	[239]
	Alginate/fibronectin	✓	—	—	[239]
	Matrigel	✓	—	—	[239]
	Fibrin	—	✓	✓	[240]
	Gellan-gum/GRGDS	✓	—	—	[190]

OECs	SAP-IKVAV	✓	—	—	[243]
	PHB-b-DEG	✓	—	—	[244]
	Alginate	*✗*	—	—	[239]
	Alginate/fibronectin	✓	—	—	[239]
	Matrigel	✓	—	—	[239]

OECs plus MSCs	Serum-derived albumin	—	✓	✓	[245]

SCs	PHB	*✗*	*✗*	✓	[249]
	PAN/PVC	—	*✗*	✓	[129]
	Alginate	✓	—	—	[239]
	Laminin/collagen	—	✓	✓	[247]

SCs plus OECs	PAN/PVC plus chABC delivery	—	✓	✓	[250]
	PLLA-PLLA oligomers	—	*✗*	✓	[251]

BM-MSC-SCs	Matrigel	✓	✓	✓	[246, 252]

✓: improvements observed; *✗*: no improvements observed; —: not studied.
